# Molecular subtype and RNA transcriptomics validation for rheumatoid arthritis characterized by fatty acid metabolism-related immune landscape

**DOI:** 10.3389/fimmu.2025.1611000

**Published:** 2025-07-24

**Authors:** Peng Zhang, Yu Wen, Xin Li, Yihong Yang, Youbang Liang, Chenguang Zhan, Liyan Mei, Haifang Du, Xiumin Chen, Maojie Wang, Runyue Huang, Xiaodong Wu

**Affiliations:** 1 The Second Clinical Medical College, Guangzhou University of Chinese Medicine, Guangzhou, China; 2 State Key Laboratory of Dampness Syndrome of Chinese Medicine, The Second Affiliated Hospital of Guangzhou University of Chinese Medicine (Guangdong Provincial Hospital of Chinese Medicine), Guangzhou, China; 3 Guangdong-Hong Kong-Macau Joint Lab on Chinese Medicine and Immune Disease Research, Guangzhou University of Chinese Medicine, Guangzhou, China; 4 The Second Affiliated Hospital, Guangzhou University of Chinese Medicine (Guangdong Provincial Hospital of Chinese Medicine), Guangzhou, China; 5 Guangdong Provincial Key Laboratory of Clinical Research on Traditional Chinese Medicine Syndrome, Guangzhou, China; 6 State Key Laboratory of Traditional Chinese Medicine Syndrome, The Second Affiliated Hospital of Guangzhou University of Chinese Medicine, Guangzhou, China

**Keywords:** rheumatoid arthritis, fatty acid metabolism, subtype classification, immune cell infiltration, RNA sequencing

## Abstract

**Background:**

Rheumatoid arthritis (RA) is a rheumatic disease charactered by severe bone destruction. Evidence suggests that fatty acid metabolism (FAM)-related proteins can regulate inflammation of synoviocytes in RA. However, the fundamental roles of FAM regulators in RA remain to be elucidated.

**Methods:**

We selected the GSE93272 dataset sourced from the Gene Expression Omnibus (GEO) for the classification of FAM-associated molecular subtypes and immune microenvironments in RA. Subsequently, bone marrow-derived macrophages (BMMs) with or without receptor activator of nuclear factor kappa-B ligand (RANKL) intervention were harvested for RNA sequencing (RNA-seq) to verify FAM hub gene expressions.

**Results:**

Difference analysis between RA samples and controls screened 53 significant FAM regulators. Random forest algorithm for RA risk prediction was utilized to identify ten diagnostic FAM regulators (hub genes). A nomogram incorporating hub genes was developed, and decision curve analysis suggested its potential utility in clinical practice. Additionally, consensus clustering analysis of these hub genes categorized RA patients to different FAM clusters (cluster A and cluster B). To quantify FAM clusters, principal component analysis (PCA) was adopted to count FAM score of every sample. ClusterB may be more linked with osteoclastogenesis in RA characterized by RXRA, IL17RA, and TBXA2R. Additionally, cases in cluster A were associated with the immunity of activated CD4 T cell, activated CD8 T cell, eosinophil, Gamma delta T cell, immature dendritic cell, MDSC, macrophage, regulatory T cell, and Type 2 T helper cell, while cluster B was linked to CD56dim natural killer cell, Natural killer T cell, T follicular helper cell, Type 1 T helper cell immunity, which has a higher FAM score. Remarkably, RNA-seq analysis confirmed the expression trend of SREBF1, FASN, CD36, SCD1 and SCD2, consistent with bioinformatics predictions.

**Conclusions:**

This scoring system of FAM subtypes provided promising markers and immunotherapeutic strategies for future RA treatment.

## Introduction

Rheumatoid arthritis (RA) is a rheumatic disease that is caused by autoimmune inflammatory factors, leading to increased susceptibility of joint swelling and stiffness, as well as pain, synovitis and cartilage damage (1). According to the current report, about 30% of RA patients develop osteoporosis in their spine or hip (2). Studies indicate that people suffering from RA account for 0.5% to 1.0% in the general population (3). To date, despite effective therapies, sustained remission in RA remains challenging, especially in difficult-to-treat cases, and approximately one-third of patients don’t respond to the recommended treatment for RA with existing medicinal products (4, 5). RA significantly threatens patients’ health and quality of life, potentially leading to disability and decreased life expectancy, which raises healthcare costs and financial burdens on families and society (6). As research related to RA continues to be conducted in depth, there is increasing evidence that RA is a complicated disease featured by substantial heterogeneity and genetic variability (7). Thus, from a genetic perspective, preliminary identification of high-risk patients for developing RA is indispensable and of great importance, as it will profoundly influence the management of RA epidemiology.

The differentiation of macrophages into osteoclasts induced by cytokines such as RANKL is the core pathological basis of bone destruction in RA, and cell metabolic reprogramming is a key link in the differentiation process of macrophages into osteoclasts (8). It has been reported that fatty acid metabolism (FAM) is an influential metabolic alteration in CD8 T cells from RA patients (9). Fatty acids act as a promising treatment choice for autoimmune disorders such as RA, which play an important role in regulating immune and non-immune pathways, potentially slowing the development of RA autoimmunity both systemically and locally (10). The rheumatoid synovial cells have the ability to derive fatty acids from both intracellular and extracellular environments, and alters FAM in immune regulation and activation of macrophages (11). Moreover, FAM-related proteins have been reported to regulate inflammation of fibroblast-like synoviocytes in RA, suggesting that FAM-related proteins hold potential as targets for use of diagnosing and treating RA (12, 13). Therefore, FAM is integral to the pathological processes of RA through the regulation of FAM-related gene expression. However, the precise functions of FAM modulators in RA remain inadequately elucidated.

**Figure 1:**
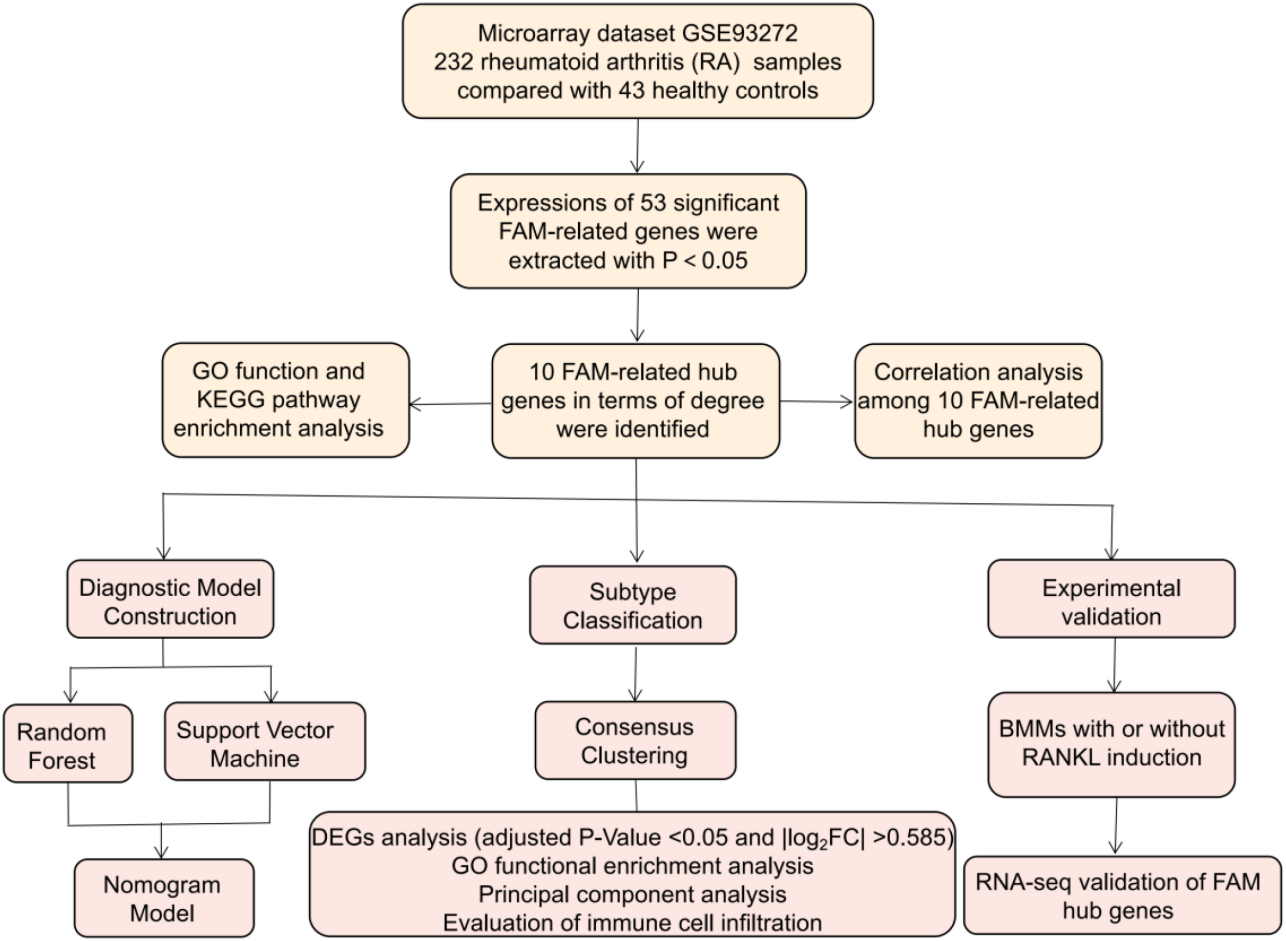
Flow chart of the study design.

In this study, the GSE93272 dataset was utilized to investigate the involvement of FAM regulators in identifying molecular subtypes and uncovering potential diagnostic biomarkers of RA. We devised gene signature for RA susceptibility, incorporating 10 key FAM regulators including SREBF1, SCD, PPARG, PPARA, INSR, FASN, CD36, ACADVL, ACADM, ACACA, and our findings revealed significant clinical benefits for patients utilizing this model. We uncovered two distinct FAM clusters strongly associated with significant immune cell infiltration, suggesting their potential diagnostic value in RA and guiding treatment decisions. Furthermore, we explored the relationships between FAM clusters and IL17RA, TBXA2R, and RXRA, which are closely related to osteoclast differentiation. The study’s design process flowchart is depicted in **Figure 1**.

## Methods

### Inclusion of eligible dataset

We retrieved the GEO platform (http://www.ncbi.nlm.nih.gov/geo/) for eligible RA data from whole blood. We used “Rheumatoid arthritis”, “Homo sapiens”, and “Expression profiling by array” as search keywords, and suitable datasets were screened based on the following criteria: the dataset includes a minimum of 80 samples comprised of downloadable raw data and series matrix files, with at least 40 samples each in the control and RA groups. After careful screening, we selected the dataset GSE93272 (14), which fully meet our criteria with 232 RA cases and 43 controls.

### Annotation and analysis for FAM-related expression profile

We adopted annotation package (R4.1.2) from Bioconductor (http://bioconductor.org/) to transform microarray probes into gene symbols. Then, the data was standardized through quantile normalization, including 232 RA samples and 43 controls. The FAM-related genes were retrieved and identified using the GeneCards database (https://www.genecards.org/) with “fatty acid metabolism” as a keyword. Totally 104 FAM-related genes (shown in **Supplementary Table 1**) were screened with a relevance score ≥50 (15) for the subsequent analysis. We used Limma package to identify differentially expressed FAM regulators between controls and RA patients. We screened the significant FAM regulators according to screening thresholds of |log_2_ fold change (FC)|>0 and *P*-Value<0.05 *(*16). Then, the R package “clusterProfiler” was used to perform GO and KEGG enrichment analysis to explore the underlying mechanism of the FAM regulators implicated in RA. Moreover, we constructed the protein-protein interaction (PPI) network of these FAM regulators by the STRING database (https://string-db.org/), conducted network topology analysis and screened the top ten targets as FAM hub genes in terms of degree through Cytoscape software (v3.8.0).

### Model construction

Two machine learning algorithms including random forest (RF) and support vector machine (SVM) models were adopted to predict the occurrence of RA. The vital FAM modulators were screened in virtue of the R package “RandomForest” when their significance scores (Mean Decrease Gini) were greater than 2. In the SVM model, the variable n signifies the count of FAM hub genes, with each data point depicted as a dot within an n-dimensional space. We then selected an optimal hyperplane that distinctly separated the control and RA groups (17). Subsequently, the “rms” R package was employed to develop a nomogram model for predicting the prevalence of RA patients based on the identified candidate FAM regulators. Calibration curves assessed the accuracy of the prediction values against actual outcomes. Decision curve analysis (DCA) was conducted to generate a clinical impact curve and evaluate whether model-based decisions were advantageous for patients (18).

### Subgroup classification

Through consensus clustering with resampling, each member and its corresponding subcluster number were identified, demonstrating the validity of the clusters (18). Using the “ConsensusClusterPlus” R package, different FAM patterns were identified based on FAM hub genes (19).

### GO enrichment analyses of DEGs between different FAM subtypes

Differentially expressed genes (DEGs) between different FAM clusters were identified using the Limma package, applying a threshold of adjusted *P*-Value <0.05 and |log_2_FC| >0.585. GO analysis was then conducted with the “clusterProfiler” R package to explore the involvement of DEGs in the process of RA (20).

### FAM score calculation

To quantify the FAM clusters, principal component analysis (PCA) was adopted to assess the FAM score for each sample. This score was calculated using the following formula: FAM score = PC1_i_, where PC1 represents principal component 1, and i indicates distinct FAM gene expression (21).

### Immune infiltration analysis

Single sample gene set enrichment analysis (ssGSEA) was used to quantify the levels of immune infiltration in RA group. Initially, the gene expression levels in the samples were ranked through sequencing using ssGSEA. Subsequently, we examined the input dataset for FAM hub genes and compiled their expression levels. From this analysis, we determined the quantity of immune cells present in each sample (22).

### Experimental animals

The Ethics Committee of Laboratory Animals in Guangdong Provincial Hospital of Chinese Medicine approved all studies. Female Sprague–Dawley(SD) rats, aged 8 weeks and weighing 200–220g, were purchased from the Experimental Animal Center of Guangzhou University of Chinese Medicine (Guangzhou, China). They were maintained under standard environmental conditions (22 ± 2°C, 50% humidity, and a 12-h light/dark cycle) with unrestricted access to food and water. The rats were euthanized under isoflurane anesthesia.

### Ethics statement

All animal experiments were approved by the Ethics Committee of Laboratory Animals in Guangdong Provincial Hospital of Chinese Medicine (No. 2023081) and conducted in accordance with the relevant guidelines. The study was carried out in compliance with the ARRIVE guidelines.

### RNA-seq analysis of bone marrow-derived macrophages with or without RANKL induction to verify differential expression of FAM genes

To isolate BMMs, we flushed long bones from 8-week-old rats using warm, serum-free alpha-minimum essential medium (α-MEM). The isolated BMMs were cultured with M-CSF (100 ng/mL) for 2 days to recruit macrophages, followed by the addition of RANKL (50 ng/mL) to induce osteoclast differentiation. RNA-seq analysis was then performed to examine the differential expression of FAM-related genes between groups with and without RANKL induction during osteoclast differentiation. Libraries from different samples were pooled according to quantitative assessments, and the final data were used for sequencing. DEGs were identified by comparing control and RANKL-induced samples using the Limma R package. FAM modulators were subsequently identified, and their expression profiles were established based on the data. The criteria for detecting FAM DEGs were set at *P* < 0.05.

### Statistical analysis

To evaluate the relationships among significant FAM genes, linear regression analyses were used. Group comparisons in the bioinformatics analysis were conducted with Kruskal-Wallis tests, and corrected t-tests were applied to assess RNA-seq data. All parametric tests were two-tailed, with *P*<0.05 deemed statistically significant. Results are shown as mean ± standard deviation.

## Results

### Retrieval of the 53 RA-related FAM genes

**Figure 2:**
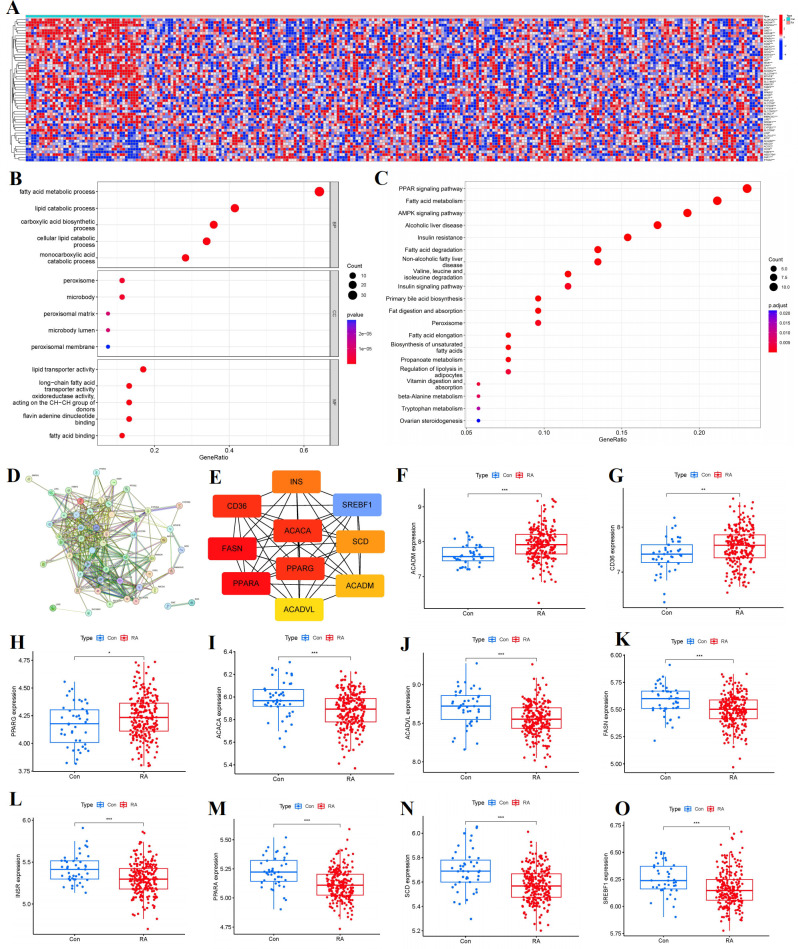
Identification of the 53 FAM modulators in RA. **(A)** Expression heat map of the 53 FAM modulators in controls and RA cases. **(B, C)** GO and KEGG enrichment analysis based on the 53 FAM modulators. **(D)** PPI network of 53 distinct FAM regulators. **(E)** The top 10 FAM hub genes in terms of degree. **(F-O)** Differential expression boxplot of 10 FAM hub genes between controls and RA cases. *p < 0.05, **p < 0.01, and ***p < 0.001.

We totally screened 53 distinct FAM regulators through difference analysis of gene expression profiles between RA group and the controls (**Figure 2A**). Our analysis revealed that GO enrichment predominantly identified entries related to biological processes (notably fatty acid metabolic process), cellular components (specifically peroxisomal matrix), and molecular function (including lipid transporter activity) (**Figure 2B**). Moreover, KEGG pathway enrichment analysis uncovered that PPAR signaling pathway and fatty acid metabolism were notably significant pathways (**Figure 2C**). The PPI network of 53 distinct FAM regulators was plotted in **Figure 2D**. We ultimately screened 10 FAM hub genes (SREBF1, SCD, PPARG, PPARA, INSR, FASN, CD36, ACADVL, ACADM, ACACA), which were shown in **Figure 2E**. We observed that the expressions of ACADM, CD36, PPARG were upregulated in RA samples in comparison with controls, but the other FAM hub genes showed opposite outcomes (**Figures 2F–O**).

### Correlation among FAM hub genes in RA

**Figure 3:**
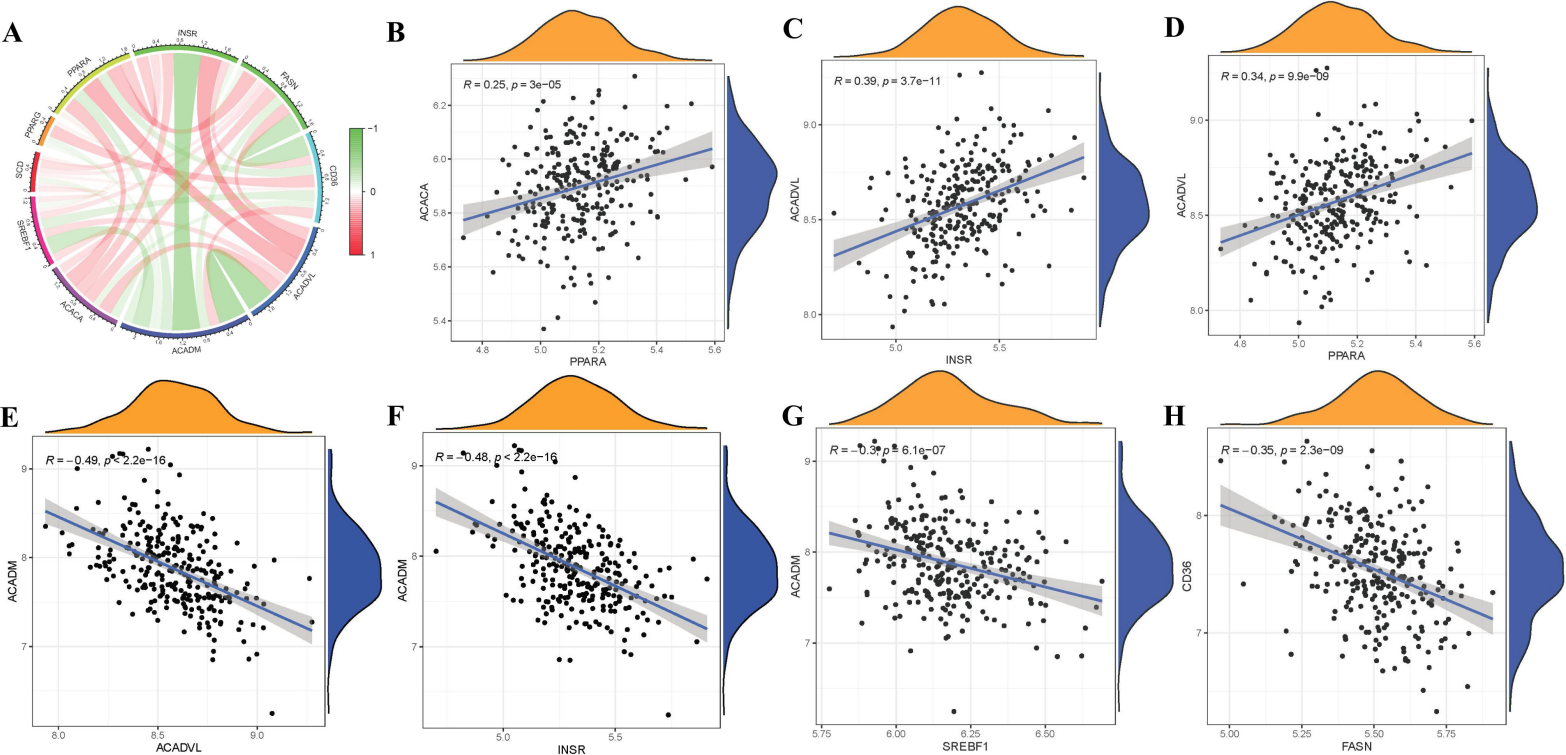
Correlation among FAM modulators in RA. **(A)** Correlation circos plot of different correlations between different FAM hub genes. There existed significantly positive correlations in the gene expression levels of ACACA-PPARA, ACADVL-INSR, ACADVL-PPARA in RA cases **(B-D)**, while the gene expression levels of ACADM-ACADVL, ACADM-INSR, ACADM-SREBF1, CD36-FASN in RA cases exhibited significantly negative correlation **(E-H)**.

To elucidate the potential correlations among significant FAM genes in RA patients, Pearson correlation analysis was conducted utilizing R statistical software. FAM hub genes in RA exhibited different relationships with each other (**Figure 3A**). Thereafter, the remarkable correlations with R>|0.25| were selected for visualization. Significantly positive relationships were observed between the gene expressions of ACACA-PPARA, ACADVL-INSR, ACADVL-PPARA in RA cases (**Figures 3B–D**), but gene expressions of ACADM-ACADVL, ACADM-INSR, ACADM-SREBF1, CD36-FASN in RA cases showed significantly negative relationships (**Figures 3E–H**).

### RF and SVM model construction

**Figure 4:**
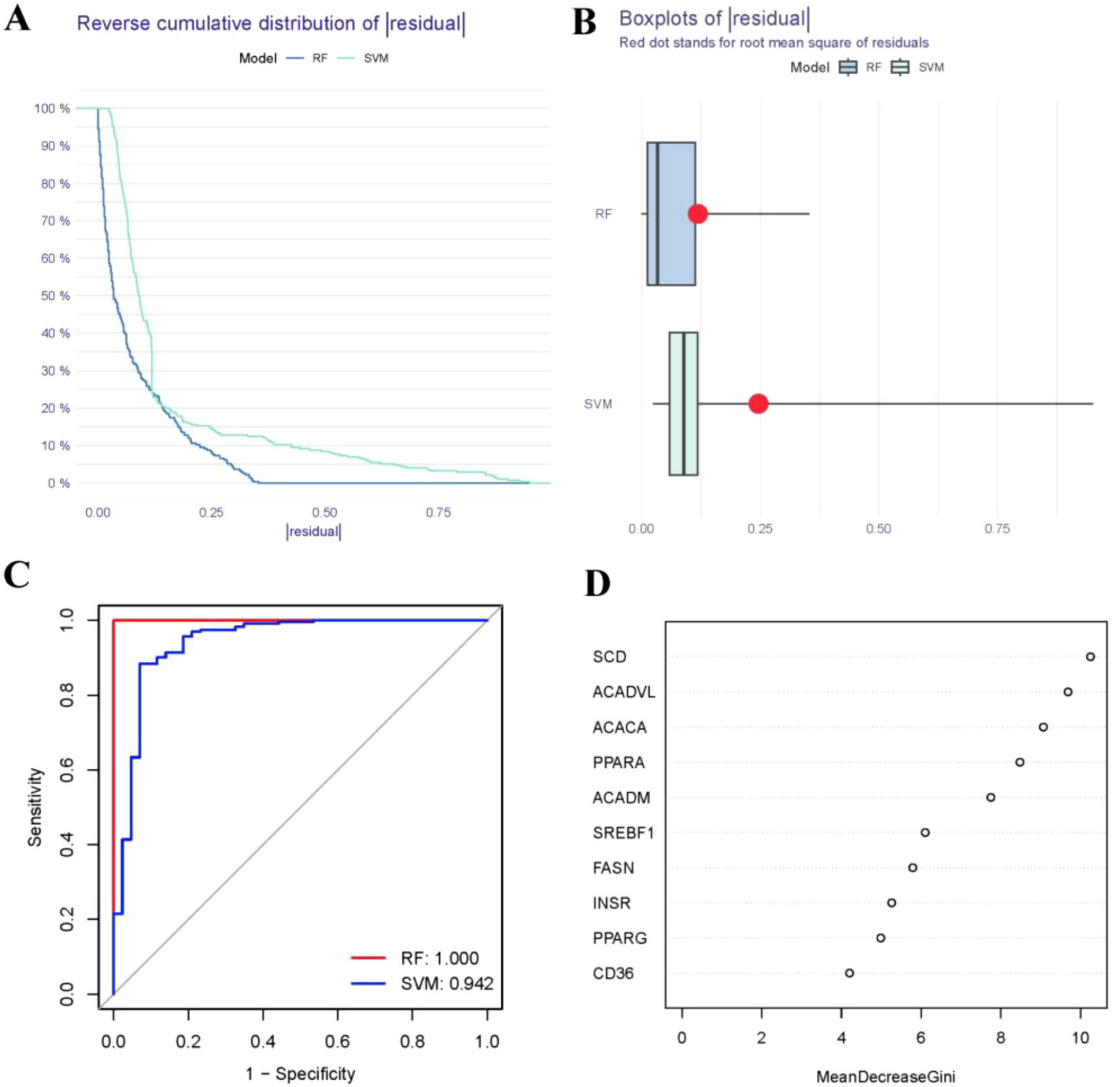
Establishment of the RF and SVM models. **(A)** Reverse cumulative distribution of residual was constructed to display the residual distribution of RF and SVM models. **(B)** Boxplots of residual was constructed to display the residual distribution of RF and SVM models. **(C)** ROC curves indicated the accuracy of the RF and SVM models. **(D)** The importance score of the 10 FAM hub genes on the basis of the RF model.

The RF model was validated to have the smaller residual according to reverse cumulative distribution of residual (**Figure 4A**) and boxplots of residual (**Figure 4B**). Most of the model samples have relatively small residuals, which indicates that the RF model is superior to the SVM model. Moreover, we utilized ROC curves to evaluate the models, and according to their AUC values, we discovered that the RF model exhibited higher accuracy than the SVM model (**Figure 4C**). As a result, we came to the conclusion that the RF model is the best one for predicting the occurrence of RA. Finally, we presented these 10 FAM hub genes based on their importance score (mean decrease Gini) and selected candidate genes with importance score>2, including SREBF1, SCD, PPARG, PPARA, INSR, FASN, CD36, ACADVL, ACADM, ACACA (**Figure 4D**).

### Construction of nomogram model

**Figure 5:**
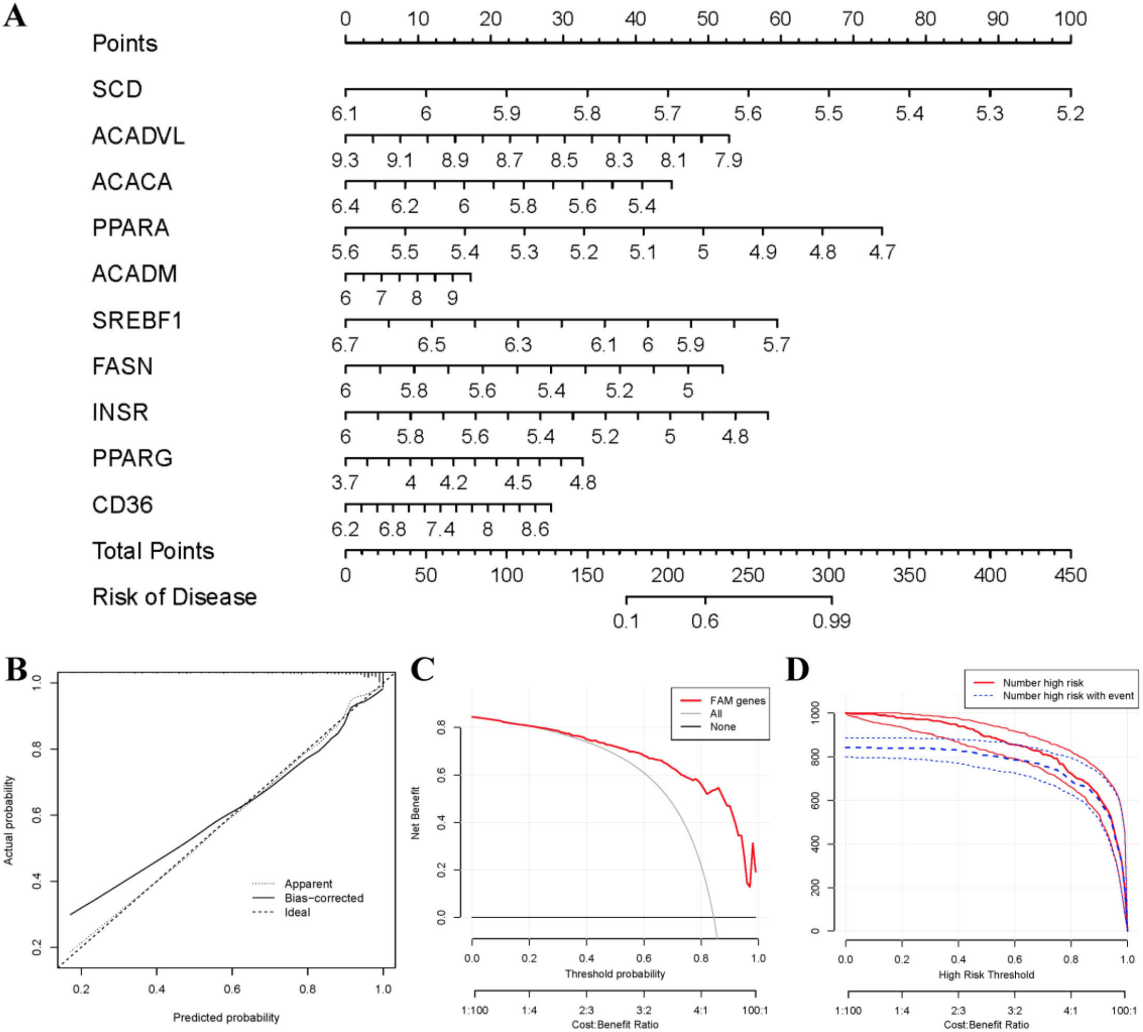
Establishment of the nomogram model. **(A)** The nomogram model was established on the basis of the 10 FAM hub genes. **(B)** The calibration curve was utilized to evaluate the predictive accuracy of the nomogram model. **(C)** Decisions on the basis of this nomogram model may be beneficial to RA patients. **(D)** The clinical impact curve was used to assess clinical impact of the nomogram model.

To predict the prevalence of RA patients, a nomogram model was constructed using the “rms” package in R based on 10 candidate FAM regulators (**Figure 5A**). The calibration curves indicated high prediction accuracy of the nomogram model (**Figure 5B**), and the DCA curve suggested potential benefits for RA patient judgments using this model (**Figure 5C**). Furthermore, the clinical impact curve demonstrated remarkable predictive capacity of the nomogram model (**Figure 5D**).

### Identification of different FAM clusters

**Figure 6:**
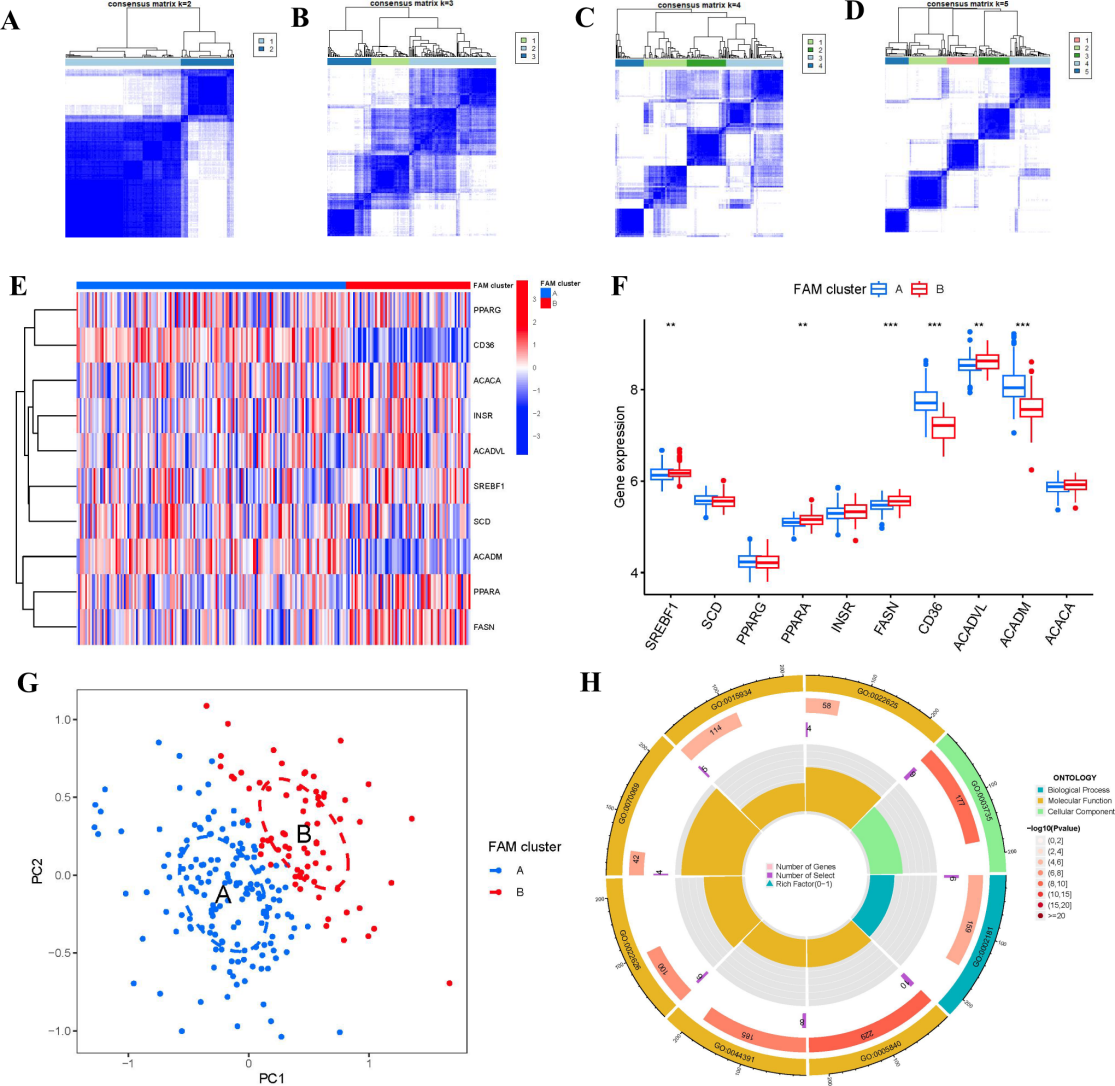
Consensus clustering of the 10 FAM hub genes in RA. **(A-D)** Consensus matrices of the 10 FAM hub genes for k = 2–5. **(E)** Expression heat map of the 10 FAM hub genes in clusterA and clusterB. **(F)** Differential expression boxplots of the 10 FAM hub genes in clusterA and clusterB. **(G)** Principal component analysis for the expression profiles of the 10 FAM hub genes that shows a remarkable difference in transcriptomes between the two FAM patterns. **(H)** GO enrichment analysis that explores the potential mechanism underlying the effect of the 74 FAM-related DEGs on the occurrence and development of RA. **p < 0.01, and ***p < 0.001.

Two FAM clusters (clusterA and clusterB) were identified on the basis of the ten FAM hub genes in virtue of the R package “ConsensusClusterPlus” (**Figures 6A–D**). Cluster A consisted of 159 samples, while cluster B included 73 samples. Subsequently, the heat map and boxplot clearly showed the differential expression levels of the 10 important FAM regulators between the two clusters. We observed that clusterA exhibited higher expression levels of CD36 and ACADM compared to clusterB, whereas SREBF1, PPARA, FASN, and ACADVL showed higher expression levels in clusterB than in clusterA. The expression levels of SCD, PPARG, INSR, and ACACA did not exhibit any noticeable variances between the two clusters (**Figures 6E, F**). The 10 FAM regulators were able to distinguish between the two FAM clusters based on the PCA results (**Figure 6G**). We identified 74 DEGs associated with FAM between the two FAM patterns. To gain further insight into the role of these DEGs in RA, we conducted GO enrichment analysis (**Figure 6H**). We observed that GO:0002181 (cytoplasmic translation), GO:0003735 (structural constituent of ribosome) and GO:0005840 (ribosome) were the mainly enriched entries.

**Figure 7:**
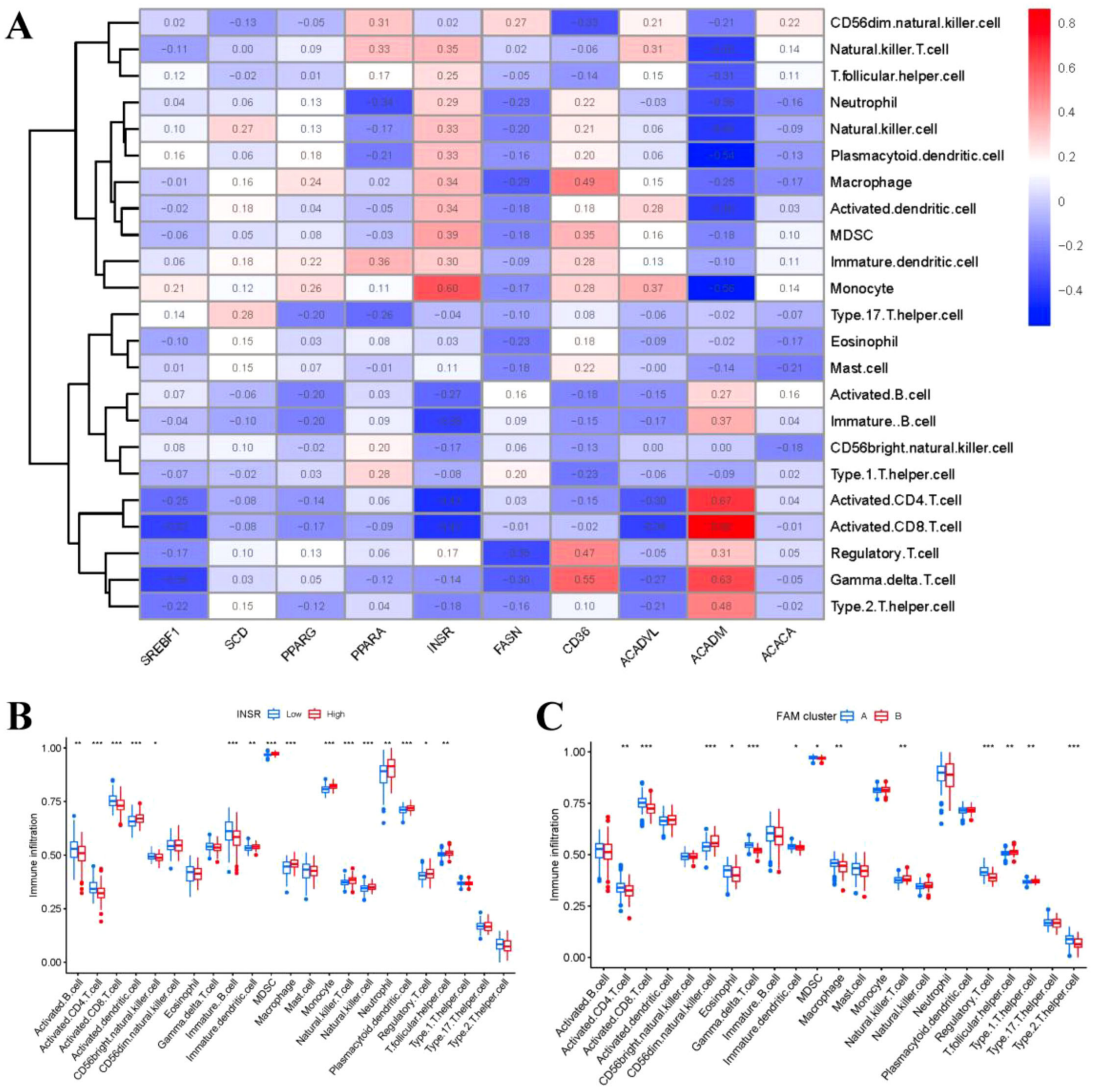
Single sample gene set enrichment analysis. **(A)** Correlation between immune cell infiltration and the 10 FAM hub genes. **(B)** Difference in the abundance of infiltrating immune cells between high and low INSR protein expression groups. **(C)** Differential immune cell infiltration between clusterA and clusterB. *p < 0.05, **p < 0.01, and ***p < 0.001.

We then explored the relationship between immune cells and 10 important FAM regulators by using ssGSEA to assess the abundance of immune cells in RA samples. We observed a positive association between INSR and multiple immune cells (**Figure 7A**). We compared the differences in immune cell infiltration between patients with high and low INSR expressions. Our results showed that patients with high INSR expression had significantly increased immune cell infiltration compared to those with low INSR expression (**Figure 7B**). Furthermore, we found that clusterA cases were associated with activated CD4 T cell, activated CD8 T cell, eosinophil, Gamma delta T cell, immature dendritic cell, MDSC, macrophage, regulatory T cell, and Type 2 T helper cell immunity; while clusterB was linked to CD56dim natural killer cell, Natural killer T cell, T follicular helper cell, Type 1 T helper cell immunity (**Figure 7C**).

### FAM gene signature construction with two gene clusters

**Figure 8:**
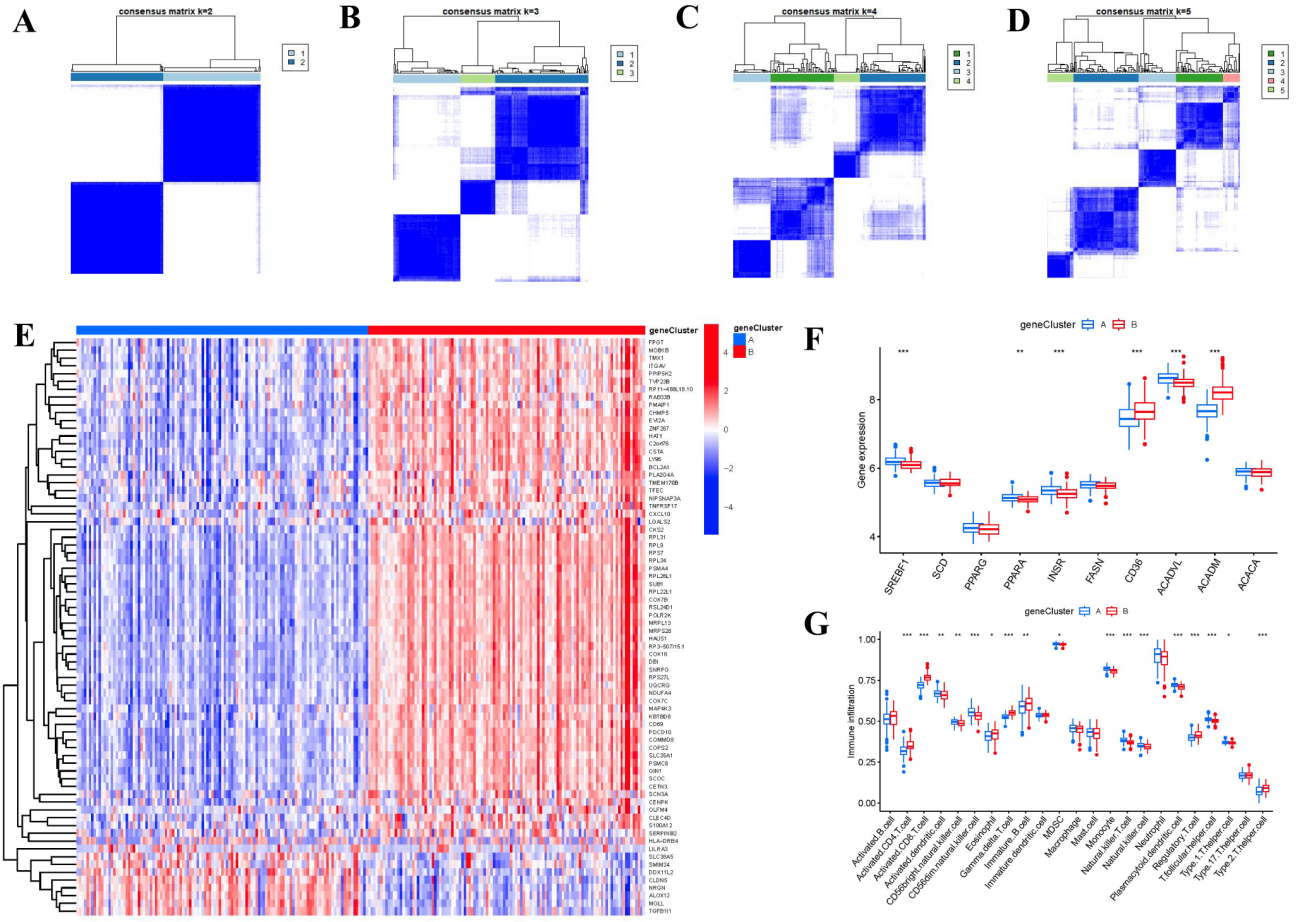
Consensus clustering of the 74 FAM-associated DEGs in RA. **(A-D)** Consensus matrices of the 74 FAM-associated DEGs for k = 2–5. **(E)** Expression heat map of the 74 FAM-associated DEGs in gene clusterA and gene clusterB. **(F)** Differential expression boxplots of the 10 FAM hub genes in gene clusterA and gene clusterB. **(G)** Differential immune cell infiltration between gene clusterA and gene clusterB. *p < 0.05, **p < 0.01, and ***p < 0.001.

Based on the 74 FAM-associated DEGs, we used a consensus clustering technique to classify RA cases into different genomic subtypes in order to understand FAM patterns. We identified two distinct FAM gene clusters (gene clusters A and B) that aligned with the sectionalization of FAM patterns (**Figures 8A–D**). The expression levels of the 74 FAM-related DEGs in gene cluster A and gene cluster B were depicted in **Figure 8E**. Similarities in immune cell infiltration levels and expressions of 10 significant FAM modulators between gene clusterA and gene clusterB also mirrored those in the FAM clusters (**Figures 8F, G**). These results once again confirmed the accuracy of our sectionalization using the consensus clustering method.

### Role of specific genes from FAM clusters for RA identification

**Figure 9:**
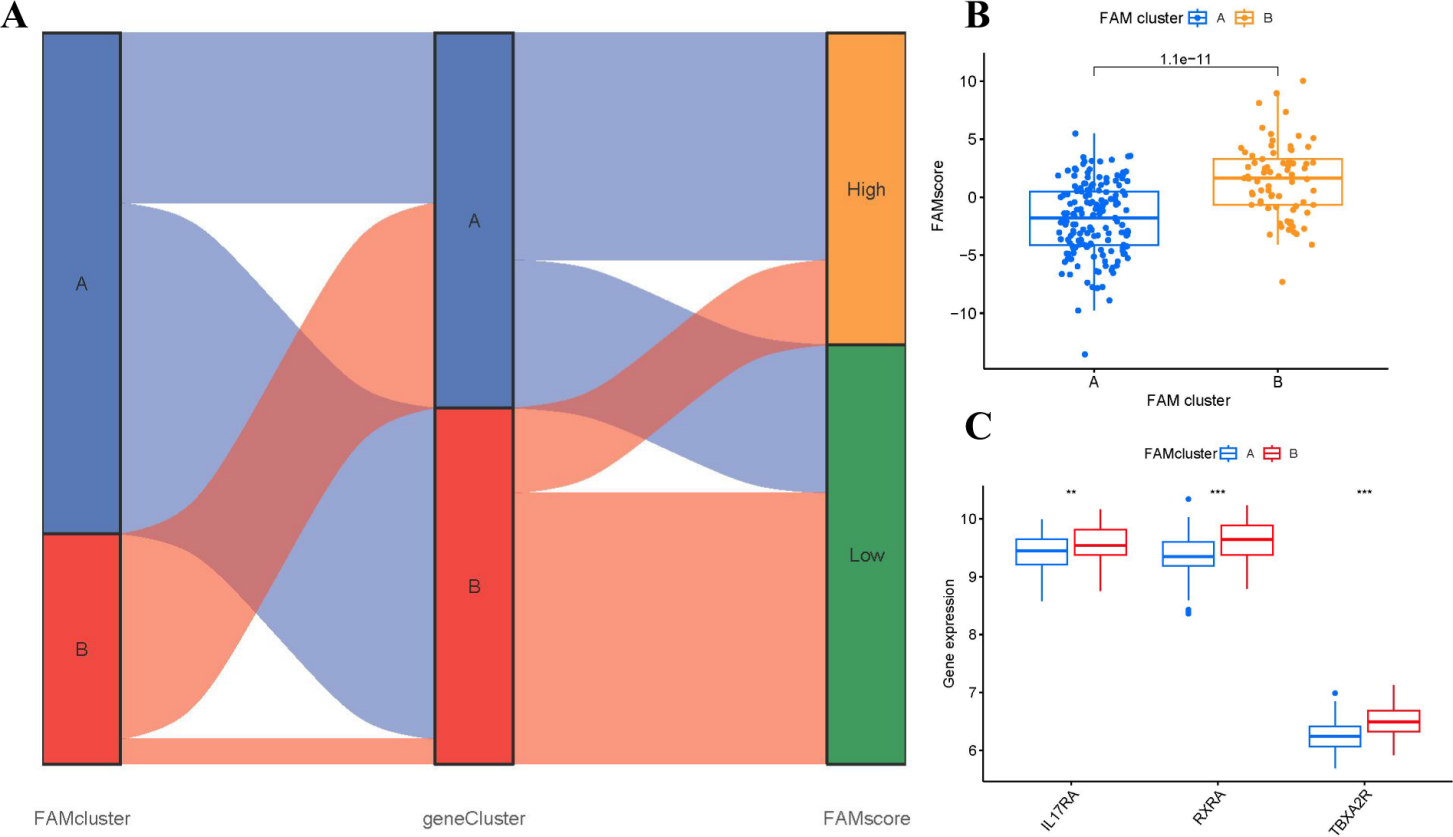
Role of FAM patterns in distinguishing RA. **(A)** Sankey diagram showing the relationship between FAM patterns, FAM gene patterns, and FAM scores. **(B)** Differences in FAM score between clusterA and clusterB. **(C)** Differential expression levels of osteoclast differentiation-related genes IL17RA, TBXA2R, and RXRA between clusterA and clusterB. **p < 0.01, and ***p < 0.001.

The Sankey diagram (**Figure 9A**) illustrated the connection between FAM scores, FAM clusters, and FAM gene clusters. PCA methods were employed to measure the FAM clusters by determining the FAM scores for each sample across the two distinct FAM clusters. In comparison to clusterA, we observed that clusterB showed a higher FAM score (**Figure 9B**). To explore the associations between FAM clusters and RA, we assessed the relationships between FAM clusters and three specific genes including IL17RA, TBXA2R, and RXRA, which have close association with osteoclast differentiation. We found that clusterB exhibited higher levels of IL17RA, TBXA2R, and RXRA expression than clusterA, indicating that clusterB may be strongly connected with RA defined by osteoclast differentiation (**Figure 9C**).

### RNA-seq validation of FAM hub genes

**Figure 10:**
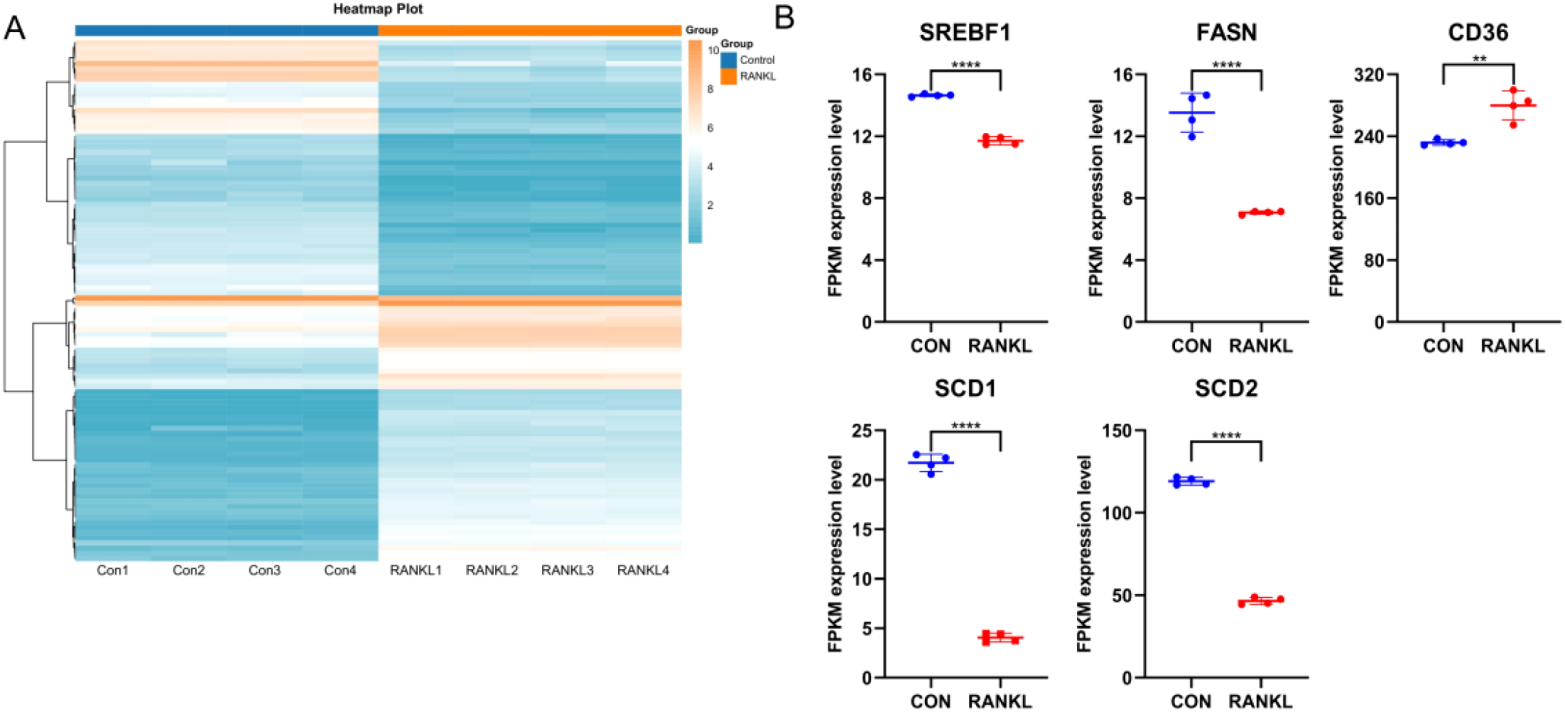
RNA-seq validation of significant FAM modulators. **(A)** Expression heat map of RANKL-induced samples and controls, assessed by RNA-seq. **(B)** The FAM modulator CD36 exhibited increased expression levels in RANKL-induced samples compared with controls, while the FAM modulators SREBF1, FASN, SCD1 and SCD2 exhibited decreased expression levels in RANKL-induced samples compared with controls. All results are expressed as means ± standard deviations. **p < 0.01, ****p < 0.0001.

The expression heat map (**Figure 10A**) showed the differential expression profiles during osteoclast differentiation. Specifically, the FAM modulator CD36 exhibited increased expression levels in RANKL-induced group compared with controls, while the FAM modulators SREBF1, FASN, SCD1 and SCD2 exhibited decreased expression levels in RANKL-induced group compared with controls (**Figure 10B**), which verified the bioinformatics results.

## Discussion

RA is a common autoimmune disorder characterized by polyarticular stiffness, synovitis, and progressive bone destruction, which may lead to irreversible disability if not managed early and effectively (23). Therefore, optimistic prognosis is strongly attributed to prompt diagnosis and effective management of RA (24). Recent studies suggest that FAM plays a key regulatory role in the inflammatory process of fibroblast-like synoviocytes, a critical cell type in RA pathogenesis (25). However, the function and importance of FAM regulators in RA remains largely unclear.

In this study, we systematically explored the expression and functional significance of FAM regulators in RA. Through differential expression analysis between RA and healthy samples, we identified 53 differentially expressed FAM-related genes and further screened 10 hub FAM regulators based on network degree values. These genes (SREBF1, SCD, PPARG, PPARA, INSR, FASN, CD36, ACADVL, ACADM, ACACA) were integrated into a predictive nomogram model based on a constructed RF model for forecasting RA occurrence, which demonstrated favorable performance in risk assessment and clinical decision-making through DCA evaluation.

More importantly, previous studies have revealed that these FAM hub genes are intricately involved in regulating bone metabolism balance in RA. For instance, sterol regulatory element binding protein 1 (SREBP1) and stearoyl-CoA desaturase (SCD), peroxisome proliferator activated receptor gamma (PPARG), peroxisome proliferator activated receptor alpha (PPARA) serving as lipogenic genes have been reported to regulate FAM progress in RA (26, 27). SREBF1 participates in reducing the activation of PI3K/AKT/NF-κB signaling pathway, which alleviates joint inflammation and bone destruction in RA model mice (28). Since increased energy consumption triggered by inflammation in RA leads to deficient FAM-related anabolic metabolism, the regulations of PPARG and SCD1 could rescue FAM homeostasis (29). PPARA agonist has been used to enhance anti-inflammatory activity in RA (30). Medium-chain acyl-CoA dehydrogenase (ACADM) has been reported to regulate fatty acid oxidation and promote lipolysis (31). Our present study indicated that acetyl-CoA carboxylase 1 (ACACA) and very long-chain specific acyl-CoA dehydrogenase (ACADVL), as the enzymes of fatty acid oxidation, were down-regulated in RA patients, as reported previously (32). High expression of cluster of differentiation 36 (CD36) has been reported to trigger inflammatory response in RA (33). Importantly, our present study has confirmed that CD36 as FAM regulators exhibited higher expression levels both in bioinformatics and *in vitro* transcriptomic validation, which in turn promote inflammatory process in RA. Abnormal expression of fatty acid synthase (FASN) results in lipid overaccumulation, which stimulates reactive oxygen species production and activates PI3K/mTOR/NF-κB signaling pathway, thereby facilitating the progression of inflammatory responses and bone erosion in RA (34). The limited fatty acid synthesis contributes to affecting RA by regulating FASN transcription subsequent to PPARG activation (35). Growing evidence has confirmed that insulin receptor (INSR) participates in regulating immune response implicated in RA (36). Above all, the dysregulations reflect the disrupted balance between fatty acid synthesis and inflammation in RA, and these mentioned FAM regulators may play a crucial role in the onset and progression of RA.

Beyond molecular alterations, we identified FAM patterns based on these hub genes that were significantly relative to abundant macrophage infiltration, which was strongly associated with osteoclastogenesis (**Figure 7C**). Numerous studies have highlighted the critical role of FAM in regulating osteoclast formation and function, primarily through interaction with specific receptors on osteoclasts, thereby affecting intracellular signaling pathways and gene expression associated with osteoclast activity (37–39). Existing study illustrates that the osteoclastogenesis of monocyte/macrophage lineage is crucial in the pathological development of RA (40). Cells of monocyte/macrophage lineage have a critical function in regulating immune balance and the development of RA (41). Monocyte/macrophage lineage differentiates into multinucleate osteoclasts, modulating osteoclastogenesis in bone metabolism (42). RXRA, IL17RA, and TBXA2R are strongly associated with osteoclastogenesis. RXRA plays a vital role in vitamin D pathway, which is involved in regulating osteoclastogenesis in bone homeostasis (43). The immunological and skeletal systems share numerous regulatory components, including the IL-17a receptor (IL17RA), whose deletion reduces the amount of osteoclast precursors and enhances bone mass (44). Existing study has confirmed that thromboxane A2 (TxA2) can directly induce osteoclastic differentiation (45). Our previous study has confirmed that TxA2 plays an important role in RA pathology through regulating synovial cell proliferation; TBXA2R, as the receptor of TxA2, could bind to TxA2 to activate the NF-κB signaling pathway and positively regulate osteoclastogenesis, whose blockage might prevent the inflammatory process from causing bone loss and bone deterioration (46). In the present study, we classified two different FAM clusters (clusterA and clusterB) on the basis of the 10 significant FAM regulators. ClusterB showed higher expressions of RXRA, IL17RA, and TBXA2R, indicating that clusterB may be associated with osteoclastogenesis characterized by RXRA, IL17RA, and TBXA2R. Furthermore, PCA techniques were employed to ascertain the FAM scores of individual samples between the two different FAM clusters in order to quantify the FAM signatures. We observed that compared with clusterA, clusterB displayed a higher FAM score.

To experimentally validate our bioinformatics findings, we utilized RANKL-induced BMMs to trigger osteoclast differentiation. Our RNA-seq-based validation showed that FAM gene CD36 showed upregulated expression levels in RANKL-induced group compared with controls, while the FAM modulators SREBF1, FASN, SCD1 and SCD2 exhibited decreased expression levels in RANKL-induced group compared with controls, which validated the bioinformatics results and previous studies. This *in vitro* validation not only supports our model but also confirms the functional relevance of these FAM regulators in osteoclastogenesis. Our research findings provide strong evidence for the involvement of FAM regulators in RA and shed new light on their role in the development of RA. This reinforces the notion that FAM modulators play a critical role in the progression of RA. In other words, focusing on these FAM-related targets could be a promising treatment strategy for managing the equilibrium between bone formation and resorption in RA. To the best of our knowledge, this study is the first to systematically characterize the immune landscape and identify molecular subtypes of RA based on FAM-related signatures.

However, several limitations should be acknowledged in this study. Although we systematically analyzed the association between FAM regulators and immune cell infiltration, and preliminarily validated the expression of key FAM-related genes through *in vitro* transcriptomic validation, the precise molecular mechanisms by which these regulators modulate RA progression remain to be elucidated. Moreover, the current findings are largely based on bioinformatics analyses; thus, in-depth *in vivo*, *in vitro*, and clinical investigations including additional disease cohorts with systemic inflammatory profiles are required to further evaluate the specificity of the FAM scoring model.

## Conclusion

Our present study generally identified 53 distinct FAM regulators and established a nomogram model of 10 FAM hub genes that accurately predicted the occurrence of RA. Then, using the 10 FAM regulators, we verified two FAM signatures and discovered that clusterB may be more linked with osteoclastogenesis in RA characterized by RXRA, IL17RA, and TBXA2R. Importantly, this study firstly displays immune landscapes and diagnostic subtypes associated with FAM progress in RA.

## Data Availability

The datasets generated and/or analyzed during the current study are available in the GEO repository, accession number: GSE93272.
