# Correction: Molecular subtype and RNA transcriptomics validation for rheumatoid arthritis characterized by fatty acid metabolism-related immune landscape

**DOI:** 10.3389/fimmu.2025.1737085

**Published:** 2025-11-07

**Authors:** Peng Zhang, Yu Wen, Xin Li, Yihong Yang, Youbang Liang, Chenguang Zhan, Liyan Mei, Haifang Du, Xiumin Chen, Maojie Wang, Runyue Huang, Xiaodong Wu

**Affiliations:** 1 The Second Clinical Medical College, Guangzhou University of Chinese Medicine, Guangzhou, China; 2 State Key Laboratory of Dampness Syndrome of Chinese Medicine, The Second Affiliated Hospital of Guangzhou University of Chinese Medicine (Guangdong Provincial Hospital of Chinese Medicine), Guangzhou, China; 3 Guangdong-Hong Kong-Macau Joint Lab on Chinese Medicine and Immune Disease Research, Guangzhou University of Chinese Medicine, Guangzhou, China; 4 The Second Affiliated Hospital, Guangzhou University of Chinese Medicine (Guangdong Provincial Hospital of Chinese Medicine), Guangzhou, China; 5 Guangdong Provincial Key Laboratory of Clinical Research on Traditional Chinese Medicine Syndrome, Guangzhou, China; 6 State Key Laboratory of Traditional Chinese Medicine Syndrome, The Second Affiliated Hospital of Guangzhou University of Chinese Medicine, Guangzhou, China

**Keywords:** rheumatoid arthritis, fatty acid metabolism, subtype classification, immune cell infiltration, RNA sequencing

Affiliation 1 “The Second Clinical Medical College, Guangzhou University of Chinese Medicine, Guangzhou, China” was erroneously given as “Guangzhou University of Chinese Medicine, Guangzhou, China”.

The original version of the article has been updated.

