# Supplementary material for: Molecular subtype and RNA transcriptomics validation for rheumatoid arthritis characterized by fatty acid metabolism-related immune landscape
Source: Front Immunol. 2025 Jul 24;16:1611000. doi: 10.3389/fimmu.2025.1611000 (PMC12328365; doi:10.3389/fimmu.2025.1611000)
Supplement: Supplementary file 1 [file Table1.docx]

**Supplementary Table 1. 104 FAM-related genes screened from GeneCards database**

| Gene Symbol | Description | Category | Uniprot ID | Gifts | GC Id | Relevance score |
| --- | --- | --- | --- | --- | --- | --- |
| SLC17A5 | Solute Carrier Family 17 Member 5 | Protein Coding | Q9NRA2 | 48 | GC06M073593 | 122.6023102 |
| GAA | Alpha Glucosidase | Protein Coding | P10253 | 53 | GC17P080101 | 121.3298569 |
| FADS1 | Fatty Acid Desaturase 1 | Protein Coding | O60427 | 46 | GC11M061799 | 110.1395721 |
| FASN | Fatty Acid Synthase | Protein Coding | P49327 | 53 | GC17M082078 | 109.2111206 |
| ACADM | Acyl-CoA Dehydrogenase Medium Chain | Protein Coding | P11310 | 50 | GC01P075724 | 100.7068481 |
| FABP2 | Fatty Acid Binding Protein 2 | Protein Coding | P12104 | 45 | GC04M119317 | 99.74272156 |
| INS | Insulin | Protein Coding | P01308 | 51 | GC11M002159 | 99.51593018 |
| LIPA | Lipase A, Lysosomal Acid Type | Protein Coding | P38571 | 53 | GC10M089213 | 95.75650787 |
| DDC | Dopa Decarboxylase | Protein Coding | P20711 | 54 | GC07M050458 | 93.50714874 |
| FABP4 | Fatty Acid Binding Protein 4 | Protein Coding | P15090 | 47 | GC08M081478 | 93.41675568 |
| FABP1 | Fatty Acid Binding Protein 1 | Protein Coding | P07148 | 47 | GC02M088122 | 86.39076233 |
| PPARG | Peroxisome Proliferator Activated Receptor Gamma | Protein Coding | P37231 | 57 | GC03P012287 | 84.8785553 |
| APOE | Apolipoprotein E | Protein Coding | P02649 | 54 | GC19P089740 | 83.65309143 |
| ACADVL | Acyl-CoA Dehydrogenase Very Long Chain | Protein Coding | P49748 | 50 | GC17P013984 | 82.78426361 |
| HADHA | Hydroxyacyl-CoA Dehydrogenase Trifunctional Multienzyme Complex Subunit Alpha | Protein Coding | P40939 | 51 | GC02M026190 | 81.96595764 |
| FADS2 | Fatty Acid Desaturase 2 | Protein Coding | O95864 | 48 | GC11P061792 | 79.87438965 |
| ALB | Albumin | Protein Coding | P02768 | 53 | GC04P073397 | 79.81551361 |
| FABP3 | Fatty Acid Binding Protein 3 | Protein Coding | P05413 | 47 | GC01M031365 | 78.88859558 |
| PPARA | Peroxisome Proliferator Activated Receptor Alpha | Protein Coding | Q07869 | 48 | GC22P046150 | 78.78233337 |
| CPT2 | Carnitine Palmitoyltransferase 2 | Protein Coding | P23786 | 53 | GC01P053196 | 75.35305786 |
| CPT1A | Carnitine Palmitoyltransferase 1A | Protein Coding | P50416 | 51 | GC11M068754 | 74.99142456 |
| MTR | 5-Methyltetrahydrofolate-Homocysteine Methyltransferase | Protein Coding | Q99707 | 52 | GC01P236795 | 74.86825562 |
| GNE | Glucosamine (UDP-N-Acetyl)-2-Epimerase/N-Acetylmannosamine Kinase | Protein Coding | Q9Y223 | 46 | GC09M036214 | 74.25733948 |
| LPL | Lipoprotein Lipase | Protein Coding | P06858 | 53 | GC08P019901 | 73.68510437 |
| CD36 | CD36 Molecule | Protein Coding | P16671 | 53 | GC07P080369 | 73.3152771 |
| FABP5 | Fatty Acid Binding Protein 5 | Protein Coding | Q01469 | 46 | GC08P081282 | 71.69562531 |
| FAAH | Fatty Acid Amide Hydrolase | Protein Coding | O00519 | 51 | GC01P046394 | 70.42773438 |
| APOB | Apolipoprotein B | Protein Coding | P04114 | 50 | GC02M020956 | 69.54392242 |
| CETP | Cholesteryl Ester Transfer Protein | Protein Coding | P11597 | 50 | GC16P056961 | 69.26702118 |
| SCD | Stearoyl-CoA Desaturase | Protein Coding | O00767 | 53 | GC10P100347 | 69.22212219 |
| LINC01672 | Long Intergenic Non-Protein Coding RNA 1672 | RNA Gene |  | 13 | GC01P007342 | 69.2089386 |
| ADIPOQ | Adiponectin, C1Q And Collagen Domain Containing | Protein Coding | Q15848 | 50 | GC03P186842 | 68.72937012 |
| LDLR | Low Density Lipoprotein Receptor | Protein Coding | P01130 | 55 | GC19P088799 | 68.46129608 |
| CYP2D6 | Cytochrome P450 Family 2 Subfamily D Member 6 | Protein Coding | P10635 | 50 | GC22M042126 | 68.30738831 |
| FA2H | Fatty Acid 2-Hydroxylase | Protein Coding | Q7L5A8 | 49 | GC16M074712 | 68.23799133 |
| LIPC | Lipase C, Hepatic Type | Protein Coding | P11150 | 50 | GC15P058410 | 68.08472443 |
| MMACHC | Metabolism Of Cobalamin Associated C | Protein Coding | Q9Y4U1 | 47 | GC01P045500 | 67.28082275 |
| CYP2C19 | Cytochrome P450 Family 2 Subfamily C Member 19 | Protein Coding | P33261 | 48 | GC10P094762 | 67.16029358 |
| ABCD1 | ATP Binding Cassette Subfamily D Member 1 | Protein Coding | P33897 | 50 | GC0XP153724 | 66.58750153 |
| CYP3A4 | Cytochrome P450 Family 3 Subfamily A Member 4 | Protein Coding | P08684 | 52 | GC07M099834 | 66.55966949 |
| PNPLA3 | Patatin Like Phospholipase Domain Containing 3 | Protein Coding | Q9NST1 | 46 | GC22P043923 | 66.16139984 |
| FABP12 | Fatty Acid Binding Protein 12 | Protein Coding | A6NFH5 | 36 | GC08M081524 | 64.98516846 |
| AMACR | Alpha-Methylacyl-CoA Racemase | Protein Coding | Q9UHK6 | 49 | GC05M033986 | 64.93276978 |
| ACADS | Acyl-CoA Dehydrogenase Short Chain | Protein Coding | P16219 | 49 | GC12P129086 | 64.67928314 |
| BAAT | Bile Acid-CoA:Amino Acid N-Acyltransferase | Protein Coding | Q14032 | 47 | GC09M101354 | 64.54626465 |
| FABP6 | Fatty Acid Binding Protein 6 | Protein Coding | P51161 | 46 | GC05P160187 | 63.87288284 |
| SMPD1 | Sphingomyelin Phosphodiesterase 1 | Protein Coding | P17405 | 53 | GC11P006390 | 63.65904617 |
| SLC27A1 | Solute Carrier Family 27 Member 1 | Protein Coding | Q6PCB7 | 44 | GC19P089098 | 63.63273621 |
| LEP | Leptin | Protein Coding | P41159 | 51 | GC07P128241 | 61.85681152 |
| MTHFR | Methylenetetrahydrofolate Reductase | Protein Coding | P42898 | 52 | GC01M011785 | 61.85425568 |
| AKR1D1 | Aldo-Keto Reductase Family 1 Member D1 | Protein Coding | P51857 | 48 | GC07P138035 | 61.79959869 |
| SLC27A2 | Solute Carrier Family 27 Member 2 | Protein Coding | O14975 | 49 | GC15P050182 | 61.72314453 |
| IL6 | Interleukin 6 | Protein Coding | P05231 | 55 | GC07P022725 | 60.5874939 |
| TNF | Tumor Necrosis Factor | Protein Coding | P01375 | 55 | GC06P111998 | 60.45607758 |
| ELOVL2 | ELOVL Fatty Acid Elongase 2 | Protein Coding | Q9NXB9 | 43 | GC06M010980 | 59.20480347 |
| PHYH | Phytanoyl-CoA 2-Hydroxylase | Protein Coding | O14832 | 49 | GC10M013277 | 58.93376923 |
| FABP7 | Fatty Acid Binding Protein 7 | Protein Coding | O15540 | 47 | GC06P122869 | 58.82136917 |
| ACOX1 | Acyl-CoA Oxidase 1 | Protein Coding | Q15067 | 50 | GC17M075941 | 58.78561401 |
| HADHB | Hydroxyacyl-CoA Dehydrogenase Trifunctional Multienzyme Complex Subunit Beta | Protein Coding | P55084 | 50 | GC02P026243 | 58.50718307 |
| LIAS | Lipoic Acid Synthetase | Protein Coding | O43766 | 47 | GC04P039512 | 57.96918106 |
| NR1H4 | Nuclear Receptor Subfamily 1 Group H Member 4 | Protein Coding | Q96RI1 | 52 | GC12P100473 | 57.47447205 |
| CYP2C9 | Cytochrome P450 Family 2 Subfamily C Member 9 | Protein Coding | P11712 | 50 | GC10P094938 | 57.12503815 |
| CYP2E1 | Cytochrome P450 Family 2 Subfamily E Member 1 | Protein Coding | P05181 | 50 | GC10P133520 | 56.85080338 |
| ELOVL4 | ELOVL Fatty Acid Elongase 4 | Protein Coding | Q9GZR5 | 49 | GC06M079914 | 56.63593292 |
| FFAR1 | Free Fatty Acid Receptor 1 | Protein Coding | O14842 | 44 | GC19P090542 | 55.82271194 |
| ALDH3A2 | Aldehyde Dehydrogenase 3 Family Member A2 | Protein Coding | P51648 | 50 | GC17P019648 | 55.69459915 |
| ALOX5 | Arachidonate 5-Lipoxygenase | Protein Coding | P09917 | 52 | GC10P045374 | 55.30757141 |
| ELOVL6 | ELOVL Fatty Acid Elongase 6 | Protein Coding | Q9H5J4 | 45 | GC04M110045 | 55.16776657 |
| FFAR4 | Free Fatty Acid Receptor 4 | Protein Coding | Q5NUL3 | 44 | GC10P093566 | 54.95315552 |
| ELOVL1 | ELOVL Fatty Acid Elongase 1 | Protein Coding | Q9BW60 | 45 | GC01M043363 | 54.88983917 |
| ELOVL5 | ELOVL Fatty Acid Elongase 5 | Protein Coding | Q9NYP7 | 47 | GC06M053267 | 54.8301239 |
| SLC10A2 | Solute Carrier Family 10 Member 2 | Protein Coding | Q12908 | 46 | GC13M103043 | 54.70742798 |
| APOA1 | Apolipoprotein A1 | Protein Coding | P02647 | 54 | GC11M116835 | 54.69607925 |
| ACACA | Acetyl-CoA Carboxylase Alpha | Protein Coding | Q13085 | 52 | GC17M037084 | 54.44872665 |
| HSD17B4 | Hydroxysteroid 17-Beta Dehydrogenase 4 | Protein Coding | P51659 | 50 | GC05P119452 | 54.39504242 |
| ABCA3 | ATP Binding Cassette Subfamily A Member 3 | Protein Coding | Q99758 | 52 | GC16M002275 | 54.38784027 |
| SLC27A5 | Solute Carrier Family 27 Member 5 | Protein Coding | Q9Y2P5 | 45 | GC19M058479 | 53.98299789 |
| FFAR2 | Free Fatty Acid Receptor 2 | Protein Coding | O15552 | 46 | GC19P089418 | 53.38329697 |
| GGT1 | Gamma-Glutamyltransferase 1 | Protein Coding | P19440 | 53 | GC22P024583 | 53.24583817 |
| GCDH | Glutaryl-CoA Dehydrogenase | Protein Coding | Q92947 | 50 | GC19P012891 | 53.22261047 |
| SLC27A4 | Solute Carrier Family 27 Member 4 | Protein Coding | Q6P1M0 | 48 | GC09P128340 | 53.10170746 |
| SLC25A20 | Solute Carrier Family 25 Member 20 | Protein Coding | O43772 | 49 | GC03M048909 | 52.70980835 |
| ABCD3 | ATP Binding Cassette Subfamily D Member 3 | Protein Coding | P28288 | 48 | GC01P094385 | 52.67074203 |
| FADS3 | Fatty Acid Desaturase 3 | Protein Coding | Q9Y5Q0 | 41 | GC11M061873 | 52.49194717 |
| BCKDHB | Branched Chain Keto Acid Dehydrogenase E1 Subunit Beta | Protein Coding | P21953 | 47 | GC06P080106 | 52.47885895 |
| ACOX2 | Acyl-CoA Oxidase 2 | Protein Coding | Q99424 | 47 | GC03M058506 | 52.27467346 |
| MTTP | Microsomal Triglyceride Transfer Protein | Protein Coding | P55157 | 49 | GC04P099563 | 51.96419144 |
| PPARD | Peroxisome Proliferator Activated Receptor Delta | Protein Coding | Q03181 | 50 | GC06P112139 | 51.9262886 |
| CYP1A2 | Cytochrome P450 Family 1 Subfamily A Member 2 | Protein Coding | P05177 | 49 | GC15P074748 | 51.87856293 |
| HNF4A | Hepatocyte Nuclear Factor 4 Alpha | Protein Coding | P41235 | 53 | GC20P044355 | 51.57343674 |
| SLC19A3 | Solute Carrier Family 19 Member 3 | Protein Coding | Q9BZV2 | 50 | GC02M227685 | 51.55614853 |
| INSR | Insulin Receptor | Protein Coding | P06213 | 58 | GC19M007112 | 51.48702621 |
| PAH | Phenylalanine Hydroxylase | Protein Coding | P00439 | 52 | GC12M102836 | 51.3288269 |
| LIPE | Lipase E, Hormone Sensitive Type | Protein Coding | Q05469 | 51 | GC19M042401 | 51.11565018 |
| CBS | Cystathionine Beta-Synthase | Protein Coding | P35520 | 54 | GC21M043053 | 51.08654022 |
| FAR1 | Fatty Acyl-CoA Reductase 1 | Protein Coding | Q8WVX9 | 46 | GC11P013668 | 51.03800964 |
| PTGS2 | Prostaglandin-Endoperoxide Synthase 2 | Protein Coding | P35354 | 53 | GC01M186671 | 51.03141403 |
| OTC | Ornithine Transcarbamylase | Protein Coding | P00480 | 50 | GC0XP038330 | 50.81932831 |
| HADH | Hydroxyacyl-CoA Dehydrogenase | Protein Coding | Q16836 | 51 | GC04P107989 | 50.74118805 |
| SREBF1 | Sterol Regulatory Element Binding Transcription Factor 1 | Protein Coding | P36956 | 52 | GC17M017810 | 50.51185608 |
| SLC22A5 | Solute Carrier Family 22 Member 5 | Protein Coding | O76082 | 51 | GC05P132369 | 50.49563217 |
| BCKDHA | Branched Chain Keto Acid Dehydrogenase E1 Subunit Alpha | Protein Coding | P12694 | 48 | GC19P089607 | 50.28062057 |
| ELOVL7 | ELOVL Fatty Acid Elongase 7 | Protein Coding | A1L3X0 | 41 | GC05M060751 | 50.2077446 |
| CYP1A1 | Cytochrome P450 Family 1 Subfamily A Member 1 | Protein Coding | P04798 | 51 | GC15M074719 | 50.06535339 |
